# Land-use impacts on crop yield: direct and indirect roles of arthropods and associated ecosystem services in European farmland

**DOI:** 10.1007/s10980-025-02117-w

**Published:** 2025-05-08

**Authors:** Roman Bucher, Péter Batáry, Julia Baudry, Léa Beaumelle, Andrea Čerevková, Enrique G. de la Riva, El Aziz Djoudi, Tara Dirilgen, Róbert Gallé, Emmanuelle Kesse-Guyot, Alison O’Reilly, Ewa Rembiałkowska, Adrien Rusch, Henrik G. Smith, Dara A. Stanley, Stuart P. M. Roberts, Werner Ulrich, Klaus Birkhofer

**Affiliations:** 1 Department of Ecology, Brandenburg University of Technology Cottbus-Senftenberg, 03046 Cottbus, Germany; 2 ‘Lendület’ Landscape and Conservation Ecology, Institute of Ecology and Botany, HUN-REN Centre for Ecological Research, Vácrátót, Hungary; 3 Faunistics and Wildlife Conservation, Department of Agriculture, Ecotrophology, and Landscape Development, Anhalt University of Applied Sciences, Bernburg, Germany; 4 Nutritional Epidemiology Research Team (EREN), Epidemiology and Statistics Research Center (CRESS), Sorbonne Paris Nord University and University Paris Cité, Inserm U1153, INRAE U1125, CNAM, 93017 Bobigny, France; 5 French National Centre for Scientific Research CNRS, UPS, Toulouse, France; 6 Institute of Parasitology SAS, Hlinková 3, 040 01 Kosice, Slovak Republic; 7 Area of Ecology, Department of Biodiversity and Environmental Management, Faculty of Biological and Environmental Sciences, University of León, 24071 León, Spain; 8 School of Agriculture and Food Science, University College Dublin, Dublin 4, Ireland; 9 Earth Institute, University College Dublin, Belfield, Dublin Ireland; 10 Department of Biology, Maynooth University, Maynooth, Co. Kildare Ireland; 11 Department of Biology & Centre for Environmental and Climate Science (CEC), Lund University, Lund, Sweden; 12 Department of Functional and Organic Food, Warsaw University of Life Sciences, Warsaw, Poland; 13 INRAE, Bordeaux Sciences Agro, ISVV, Villenave d’Ornon, SAVE France; 14 Agroecology Lab, Université Libre de Bruxelles (ULB), Boulevard du Triomphe CP 264/02, B-1050 Brussels, Belgium; 15 Department of Ecology and Biogeography, Nicolaus Copernicus University, Toruń, Poland

**Keywords:** Agroecology, Ecological intensification, Meta-analytic structural equation models, Pollination, Pest control, Trait-based approach

## Abstract

**Context:**

Land-use intensification to increase yields is often detrimental to biodiversity undermining the provision of ecosystem services. However, it is questionable if ecosystem service providers contribute to ecological intensification by achieving the same or higher yields than conventional high-intensity agriculture.

**Objectives:**

In this study, we aimed to disentangle the effects of local and landscape-scale land-use intensification on arthropod communities and their contribution to ecosystem services and crop yield. A set of meta-analytic structural equation models allowed us to assess direct and indirect relationships in the cascade from land use to yield.

**Methods:**

We selected 37 datasets containing information on land use, community composition, levels of pollination and natural pest control services, and crop yield. We quantified functional diversity of communities by collecting trait information for three exemplary groups of service-providers: bees, ground beetles, and spiders.

**Results:**

Local land-use intensification reduced the abundance of all arthropod groups. Spiders were the only group whose species richness was negatively related to a higher percentage of arable land in the landscape. High abundance of bees related positively to oilseed rape pollination and crop yields. In the models for the two predator groups, crop yield was strongly determined by land use, independent of the pest control services provided by natural enemies.

**Conclusions:**

Our results suggest a potential for ecological intensification mediated by land-use change in crops where pollination benefits yield, but suggest more nuanced effects for pest control. Our study also calls for experiments on multiple taxonomic groups and ecosystem services that apply comparable methods at similar scales.

**Supplementary Information:**

The online version contains supplementary material available at 10.1007/s10980-025-02117-w.

## Introduction

High-intensity conventional agriculture is important for global food production. However, increases in land-use intensity are often detrimental to the environment, threatening biodiversity and associated ecosystem functions (Hooper et al. 2012). Impaired ecosystem functions could undermine the sustainable productivity of agroecosystems through reduced supporting and regulating ecosystem services (Cardinale et al. 2012; de la Riva et al. 2023). It remains unclear to what extent agroecological practices, which aim to promote supporting and regulating ecosystem services in agricultural landscapes, can contribute to crop yields when land-use intensity is reduced to diminish collateral damage to nature (Bommarco et al. 2013; Kleijn et al. 2019).

Over the last century, land-use intensification at all scales has shaped European farmland with profound impacts on landscapes and biodiversity (Tscharntke et al. 2005; Gámez-Virués et al. 2015; Hallmann et al. 2017). High-intensity local management, such as intensive tillage and application of pesticides, can directly lead to population declines and local extinctions of disturbance-sensitive species (Geiger et al. 2010; Martin et al. 2020). In addition, ploughing, harrowing, fertilizer and herbicide application can indirectly reduce species richness at higher trophic levels due to their impact on primary producers that are potential food sources or contribute to suitable vegetation structure and microclimatic conditions (Kleijn et al. 1997; Farooq et al. 2022). Organic farming is seen as an alternative, by reducing local land-use intensity by avoiding synthetic pesticides and inorganic fertilizers and therefore using more complex crop rotations to support biodiversity and ecosystem services at the farm level (Bengtsson et al. 2005; Muneret et al. 2018). In addition, there is growing evidence that land use in the surrounding landscape also plays a crucial role for animal biodiversity and that the local management extends into adjacent fields (Kleijn et al. 1997; Tscharntke et al. 2021). For example, declines in arthropod biomass, abundance, and species richness have been attributed to a higher agricultural land cover in addition to local land-use intensity (Seibold et al. 2019). Therefore, studies investigating land use and biodiversity relationships as well as biological conservation efforts ideally consider both local land-use intensity and landscape-level variables (Tscharntke et al. 2012).

Several lines of evidence suggest that there is a positive relationship between biodiversity and ecosystem services (Hooper et al. 2005; Cardinale et al. 2012). However, intertwined aspects of community structure have been found to influence ecosystem functioning. Firstly, the sheer number of individuals or amount of biomass can contribute to ecosystem resilience or improve functioning (i.e. mass ratio hypothesis; Grime et al. 1988). Secondly, a high number of species can enhance ecosystem functioning if they differ in their functional characteristics (Blüthgen and Klein 2011). For example, organisms using different resources or active at different times are expected to enhance ecosystem functioning (i.e. complementarity hypothesis; Tilman et al. 1996). However, species that share the same functional characteristics are considered to be redundant from a purely functional perspective, but still contribute to the resilience of functions in the event of major environmental changes (see insurance hypothesis; Loreau et al. 2001). Many ecologists have suggested that a functional approach is likely to improve our understanding of the relationships between biodiversity and ecosystem functioning and enhances our ability to predict the consequences for the provision of ecosystem services (Hooper et al. 2005; Violle et al. 2007). Functional diversity is a measure of variation in the characteristics of individual organisms (i.e. traits) in a community, with the assumption that greater trait variation improves ecosystem functioning. Trait-based approaches are likely to reveal more general patterns because they can be applied across wider biogeographical areas. Empirical evidence on the relationships between abundance, species richness, and functional diversity with ecosystem services shows predominantly positive correlations (see Harris on et al. 2014 for a review), with some support that functional diversity may be a better predictor than species-based indices (Gagic et al. 2015).

Arthropods are a highly diverse taxonomic group, and many species are involved in vital ecosystem services in temperate farmland, such as biological control of pests and weeds, or pollination of flowering crops (Birkhofer et al. 2024; Garibaldi et al. 2013). Arthropods are also a functionally heterogeneous group that vary widely in many traits. For example, the dispersal ability of arthropods determines the spatial scale that is relevant to their abundance (Schmidt et al. 2008). Thus, the response of different arthropod groups to land-use intensity at local and landscape scales is expected to vary between species and functional groups (Karp et al. 2018; Martin et al. 2019). Similarly, relationships between biodiversity and ecosystem services are likely to differ between taxonomic and functional groups: While pollination is mostly beneficial for flowering plants, predation of natural enemies is not limited to pests, but often include other predators, which is expected to weaken top-down control (Rosenheim et al. 1995). Consequently, the role of arthropods in responding to land-use intensity and in affecting ecosystem services, is likely to differ between different functional groups (see Harrison et al. 2014). However, the role of arthropod functional diversity in these relationships still remains understudied (Wong et al. 2019).

To date, few empirical studies have examined how agricultural yields are affected by land-use intensity through a combination of direct effects and indirect effects mediated by ecosystem service providers (but see Gagic et al. 2017). Furthermore, the extent to which increases in ecosystem services actually contribute to crop productivity is often unknown. Single-link approaches (e.g. correlations between land use and biodiversity or between biodiversity and ecosystem services) are not suitable for quantifying the relative importance of the direct effects of land use on yield versus the indirect effects via biodiversity and ecosystem services (Ulrich et al. 2023). Where structured path analyses have been used, they have been technically limited to individual studies (Jak 2015), with limited ability to generalize results. We are not aware of any synthesis study using meta-analytic structural equation models to address the land use—biodiversity—ecosystem services—yield cascades, quantifying the direct effects of land-use intensity on yield and the indirect contribution of biodiversity via ecosystem services.

We present a pan-European meta-analysis that quantifies the direct effects of local land-use intensity on crop yield, as well as the effects of local and landscape-level land use on arthropods in the fields, crop-related regulating ecosystem services, and crop yield. We calculated separate models for the abundance, species richness, and functional diversity of three important providers of pollination and pest control services: Bees (Apoidea), ground beetles (Carabidae), and spiders (Araneae). Thereof, we compared the roles of mass ratio and species/trait complementarity hypotheses for the effects of land use on ecosystem services and crop yields. We expected positive direct effects of local land-use intensity on crop yields. In contrast, high land-use intensity at the farm and the landscape scale is expected to reduce arthropod abundance, species richness and functional diversity in crop fields. Higher abundance, species richness, and functional diversity of these arthropod groups should improve pollination and pest control, which in turn should contribute to crop yield. This could also be done directly by predatory arthropods via beneficial effects beyond the quantified pest control (e.g. if ground beetles and spiders control pests that were not monitored in the respective studies).

## Methods

### Data identification and screening

**Fig. 1:**
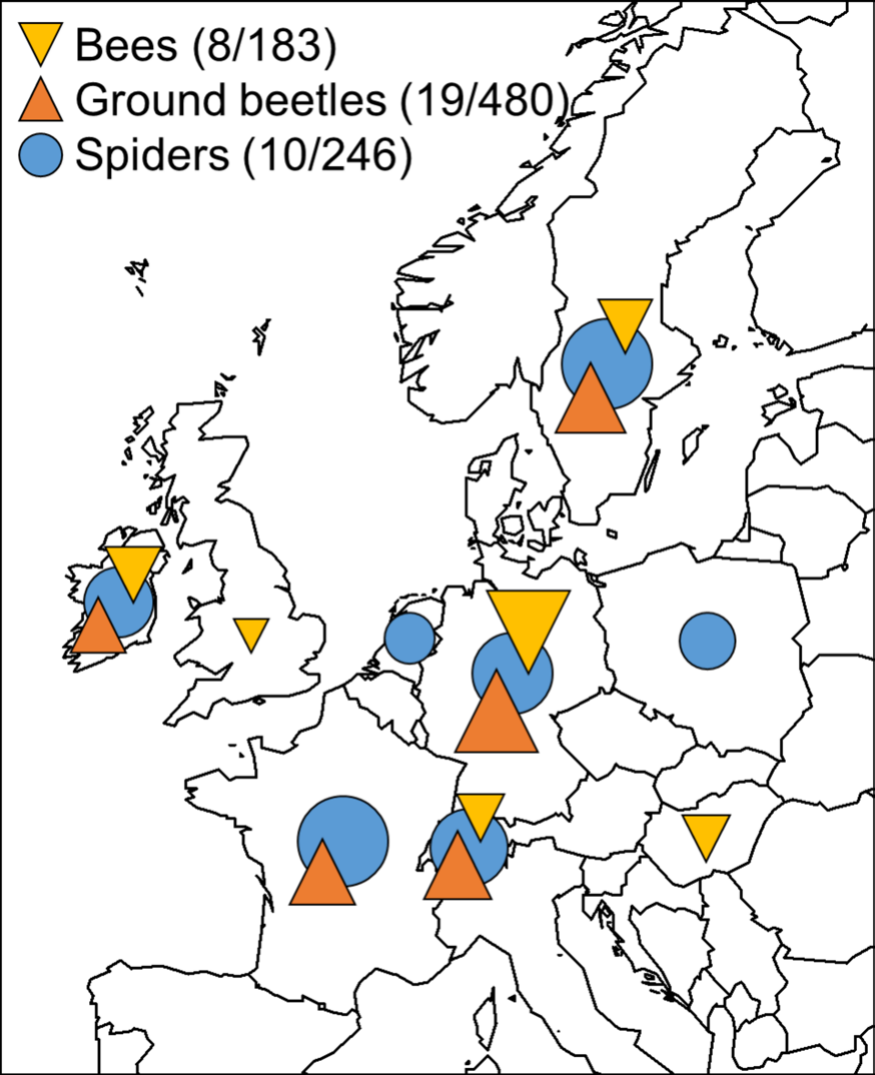
Map showing the distribution of datasets among European countries. Coloured symbols indicate the taxonomic group (first number gives the number of datasets). The size of the symbols is proportional to the number of sites per taxonomic group (second number in the legend)

Datasets were compiled from the database of an international consortium of experts in environmental change, agroecology, ecosystem services, and human health as part of an EU synthesis project (Biodiversa—FunProd). Criteria for data selection were the availability of (i) information on local and/or landscape-level land-use information, (ii) in-field community data, (iii) quantification of ecosystem services, and (iv) yield data. While the availability of species-level community composition data was mandatory, not all studies quantified all target variables. Initially, the consortium identified 60 datasets that met the above criteria. As the datasets collected largely lacked information on the traits of the species involved, which is required for the calculation of functional diversity, we were limited in the number of taxonomic groups that could be included as representatives of ecosystem providers in the re-analysis of the studies. Three arthropod groups were selected: bees, ground beetles, and spiders, as they were well represented in the datasets covering Europe (Fig. 1) and are highly relevant in agroecosystems either as pollinators or as pest control agents (Birkhofer et al. 2024; Cardoso et al. 2024). Focusing on these three arthropod groups reduced the number of suitable datasets to 53. To further reduce the heterogeneity among included studies, we decided to focus on annual crops such as wheat, barley, and oilseed rape by excluding data originating from grasslands or perennial cultivations such as vineyards or fruit orchards, resulting in a final number of 37 datasets (see Supplementary Table T1).

For the majority of selected datasets, local land-use intensity was represented by the contrast between organic and conventional farming. For datasets with multiple numerical local land-use variables (e.g. fertilizer, herbicide, and insecticide applications), the variables were divided by the arithmetic mean of the respective variable across all sites and then summed into a land-use intensity index per site (see Blüthgen et al. 2012). For our focus on European croplands, we used the percentage of arable land in the vicinity as a measure of landscape-level land-use intensity. This choice was motivated by an extensive dataset that was included in our selection, for which we had no other way to quantify other landscape-level variables (due to missing coordinates) than the percentage of arable land within a 1000 m radius provided by the primary study (Emmerson et al. 2016). For all the remaining datasets, the proportion of arable land within a 1000 m radius around the centre of the study sites was extracted from CORINE land cover data (Büttner 2014) using QGIS (2023). The selection of ecosystem service variables (e.g. seed set, aphid removal, pest abundance) and information on yield (e.g. grain and straw mass) for the reanalysis was discussed in the expert consortium. Either the most appropriate variable provided by the primary study was selected or similar aspects of an overarching variable (e.g. the abundance of different pest organisms in a single pest abundance index) were combined per study site as described above for the local land-use intensity. All variables were subsequently normalized by scaling the data values to a range between zero and one per study (if no numerical local land-use variable was available, organic field were classified with the lowest land-use intensity of zero vs. conventional farming with the maximum land-use intensity of one). An overview of the included land use, ecosystem service, and yield variables is provided as metadata for each included dataset (study-level: Supplementary Table T1; site-level: Supplementary Table T2).

### Integration of trait information

For bees, we had access to co-author Stuart Roberts’ trait database. Here, we used intertegular distance, nesting type, lecty, pollen transportation mode, and tongue length guild (Table 1). For ground beetles, we collected information on body size, mobility, trophic position, stratum preference, and breeding type (Table 1). For spiders, we collected trait information on body size, mobility, stratum preference, and hunting mode from multiple sources (Table 1). We standardized the binary coded traits so that trait values across trait categories (e.g. soil/ground/vegetation) summed up to one for each trait (stratum in this example). We used the *gawdis* function in R to group and weight trait categories to equalize the influence of each trait on species similarity, regardless of the number of categories per trait (see de Bello et al. 2021). Based on the Gower distance matrix, we calculated Rao Quadratic Entropy (hereafter Rao) as a measure of functional diversity. Rao is the average of the dissimilarity between each pair of species in a local community, weighted by the abundances of both species. There is growing consensus that Rao can be used to partition diversity across scales (de Bello et al. 2010).

**Table 1:** Detailed information on the traits included for the three arthropod groups: Identity of trait levels, quality of trait variables, and the respective literature sources for each trait

Group	Trait	Trait value/levels	Quality	Source
Bees (Apoidea)	Intertegular distance	[mm]	Numerical	Stuart Roberts
	Nesting type	Carder, excavator, mason, parasite, renter	Categorical	
	Lecty	Monolectic, mesolectic, oligolectic, polylectic	Categorical	
	Pollen transportation	Body, legs, legs and abdomen, crop, accidental transport	Categorical	
	Tongue length	Long, short	Categorical	
Ground beetles (Carabidae)	Body size	1 mm–12 mm, 12 mm–24 mm, > 24 mm	Categorical	Benisch (2023), Lompe (2023)
	Phenology	Spring breeding, autumn breeding	Categorical	Lindroth (1985)/86, Turin (2000)
	Mobility	Macropterous, brachypterous	Categorical	Lindroth (1985)/86, Turin (2000)
	Trophic position	Predator, herbivore	Categorical	Lindroth (1985)/86, Turin (2000)
	Stratum	Endogeic, epigeic, vegetation	Categorical	Luka et al. (2009)
Spiders (Araneae)	Body size	1 mm–6 mm, 6 mm–11 mm, > 11 mm	Categorical	Nentwig et al. (2024)
	Mobility	Ballooning, non ballooning	Categorical	Blandenier (2009)
	Hunting mode	Cursorial, web building	Categorical	Cardoso et al (2011)
	Stratum	Endogeic, epigeic, vegetation	Categorical	Maurer & Hänggi (1990)

### Statistical analysis

Structural equation models (SEM, also referred to as ‘path analysis’) allow the assessment of an a priori defined causal structure with direct and indirect links based on correlation and covariance matrices of empirical data from a single study by testing for conditional dependence between variables (Shipley 2016). In order to incorporate data from many studies, researchers face the challenge that studies differ in the completeness of the variables quantified. Meta-analytic structural equation models (metaSEM) address this challenge in two steps. First, multigroup SEM is used to pool the correlation coefficients. Second, the structural model is fitted to the pooled correlation matrix, using weighted least squares estimation (Cheung and Chan 2005). This ensures that associations quantified in more studies receive more weight in the estimation of the model parameters.

**Fig. 2:**
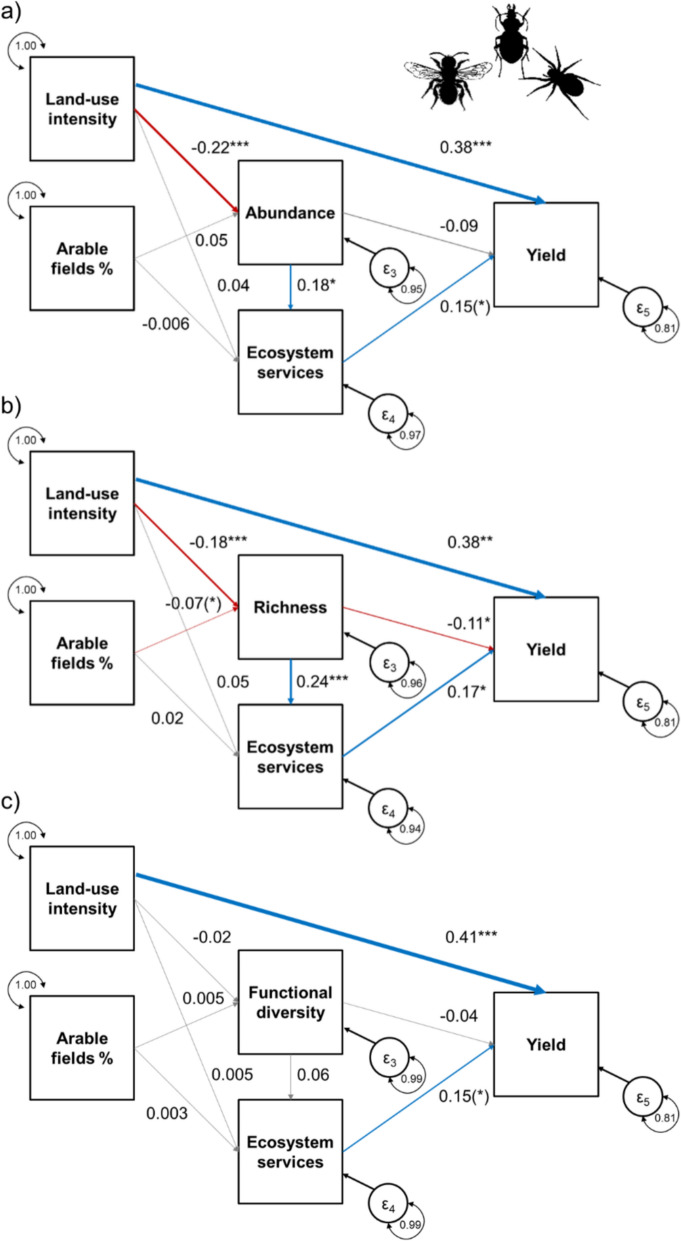
Effects of local land-use intensity and the percentage of arable land in the landscape on **a** Abundance, **b** Species richness, and **c** Functional diversity across taxa as well as on ecosystem services. The models illustrate to which extent the three biodiversity metrics influence pollination and pest control and how these ecosystem services relate to crop yields. Furthermore, the models illustrate direct impacts of local land-use and the three biodiversity metrics on crop yield. Blue arrows indicate positive, red arrows indicate negative, and grey arrows indicate non-significant relationships (p > 0.1). The width of arrows indicates the strength of the relationships based on standardized correlation coefficients (values provided next to the arrows; *** p < 0.001, ** p < 0.01, * p < 0.05, (*) p < 0.1)

In preparation for the metaSEMs, we compiled pairwise correlation coefficients between the two explanatory variables local land-use intensity and the percentage of arable land within a 1000 m radius, the three biodiversity-related metrics (abundance, species richness or functional diversity), ecosystem services (crop pollination or pest control), and crop yield, as response variable only. From this list, we created correlation matrices for each study in three versions, including either (1) abundance, (2) species richness, or (3) functional diversity as the arthropod metric. Differences in the relationships between the variables may be due to many factors (e.g. spatio-temporal differences, differences in the methods used to quantify the variables, etc.). Random effects metaSEMs are able to deal with heterogeneity in correlation coefficients by accounting for study-level variance. We used the *tssem1* function from the R-package *metaSEM* (Cheung and Chan 2005) to fit the first step of a random effects model. In the second step, we first defined the causal structure for our models by specifying all regression coefficients and all variances/co-variances in the model (see Jak 2015). Each model included the following direct links: (a) links from the explanatory variable local land use to the response variables arthropod metric, ecosystem service and crop yield, (b) links from the explanatory variable landscape-level land use to the response variables arthropod metric and ecosystem service, (c) links from arthropod metric to ecosystem service and crop yield, and (d) links from ecosystem service to crop yield that served as response variable only (see e.g. Figure 2a). We used the *tssem2* function from the R-package *metaSEM* (Cheung and Chan 2005) to fit the hypothesized model to the data. In a third step, we split the models into the three taxonomic groups to test for relationships that were idiosyncratic for each taxonomic group and ecosystem service (i.e. pollination in studies with bees and pest control in studies with either ground beetles or spiders). The output provides estimates of standardized coefficients as a measure of effect size (i.e. one standard deviation increase in the explanatory variable will lead to an increase of the respective standardized correlation coefficient in the response variable, see standardized correlation coefficients in the figures) and test statistics (z- and p-values in the tables) for all specified relationships. As a goodness-of-fit index for metaSEMs including either abundance, species richness, or functional diversity, we report root mean square errors of approximation (RMSEA) assessing the fit of the model to the observed data.

## Results

### Overall model across taxonomic groups

Higher local land-use intensity strongly increased crop yields regardless of the arthropod metric included in the model (Table 2, Fig. 2a–c). Increasing local land-use intensity reduced arthropod abundance and richness, but not functional diversity and ecosystem services. The proportion of arable land in the surrounding landscape tended to reduce species richness, but abundance and functional diversity were not significantly influenced. The proportion of arable land in the landscape was not significantly related to ecosystem services. Arthropod richness, and to a lesser extent, abundance enhanced ecosystem services. Species richness was negatively correlated with yields, whereas abundance and functional diversity were not related. In the model including species richness, we found a significant positive relationship between ecosystem services and yield, while in the models including abundance and functional diversity, higher ecosystem service values only tended to be positively correlated with crop yields. All three models achieved a satisfactory fit below the commonly used RMSEA threshold of 0.08 (see Jack 2015; Abundance: RMSEA = 0.031; Richness: RMSEA = 0.027; Functional diversity: RMSEA = 0.028).

**Table 2:** Test statistics (z- and p-values) for each quantified relationship in the metaSEM for all three arthropod groups separated by arthropod metrics (see Fig. 2 for standardized coefficients). Note that combinations of explanatory and response variables that were not included in the a priori structure are blank

Across taxa (n = 909)	Explanatory variable	Arthropod [Metric]	Ecosystem services	Yield
Abundance	Local land-use intensity	z = − 4.593, p < 0.001	z = 0.584, p = 0.589	z = 3.771, p < 0.001
	Arable fields %	z = 1.165, p = 0.244	z = − 0.103, p = 0.918	
	Arthropod [Metric]		z = 2.263, p = 0.024	z = − 1.426, p = 0.154
	Ecosystem services			z = 1.829, p = 0.067
Richness	Local land-use intensity	z = − 3.680, p < 0.001	z = 0.632, p = 0.527	z = 3.644, p < 0.001
	Arable fields %	z = − 1.664, p = 0.096	z = 0.291, p = 0.770	
	Arthropod [Metric]		z = 4.071, p < 0.001	z = − 1.968, p = 0.049
	Ecosystem services			z = 1.982, p = 0.048
Functional diversity	Local land-use intensity	z = − 0.498, p = 0.618	z = 0.059, p = 0.953	z = 4.085, p < 0.001
	Arable fields %	z = − 1.406, p = 0.160	z = 0.058, p = 0.954	
	Arthropod [Metric]		z = 0.999, p = 0.318	z = − 0.841, p = 0.400
	Ecosystem services			z = 1.741, p = 0.082

### Bees

**Fig. 3:**
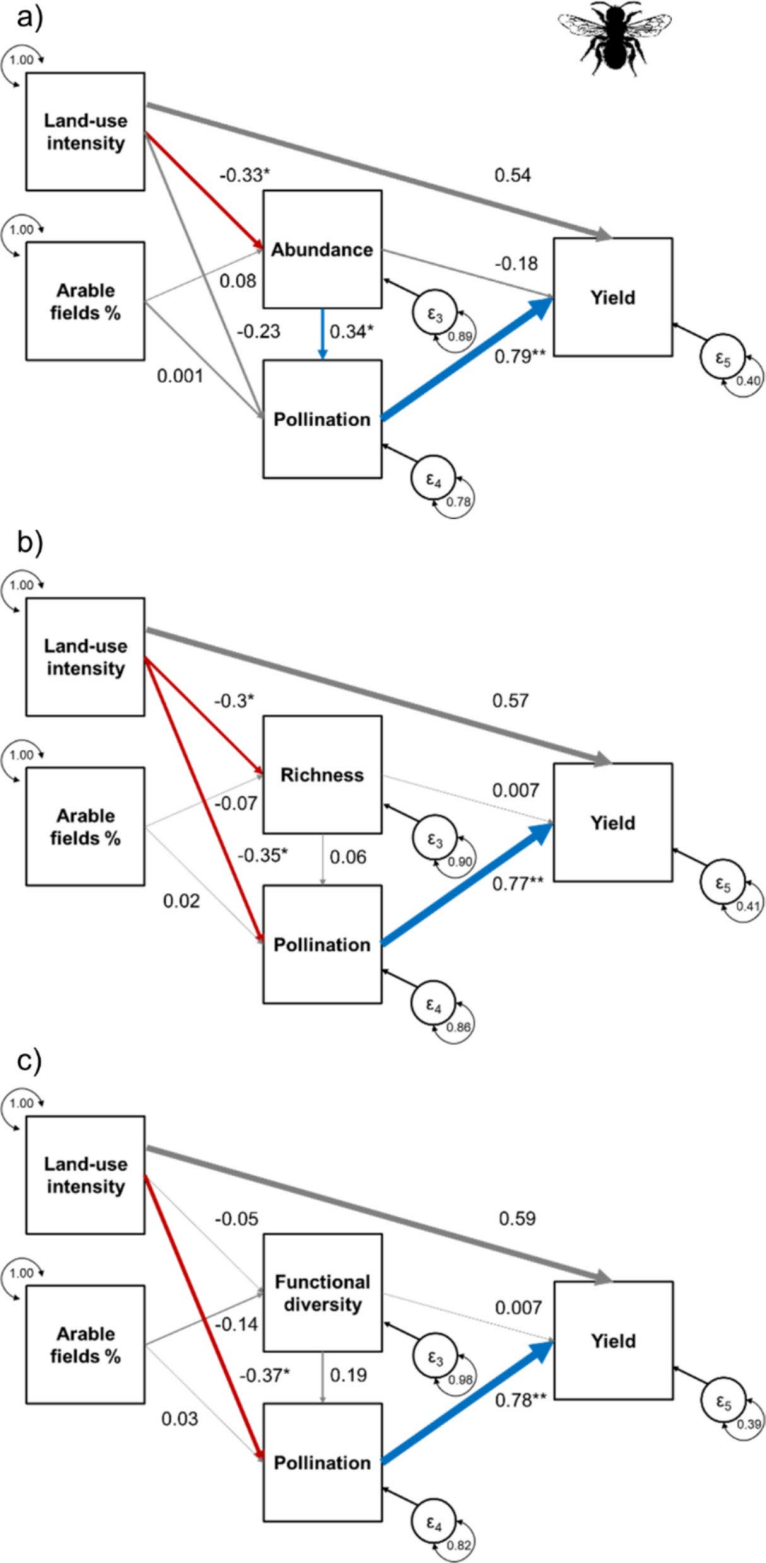
Effects of local land-use intensity and the percentage of arable land in the landscape on bees. Biodiversity metrics of bees include **a** Abundance, **b** Species richness, and **c** Functional diversity. The models illustrate to which extent the three calculated biodiversity metrics influence pollination and how this ecosystem service relates to crop yields. Furthermore, the models illustrate direct impacts of local land-use and the three biodiversity metrics on crop yield. Blue arrows indicate positive, red arrows indicate negative, and grey arrows indicate non-significant relationships (p > 0.1). The width of arrows indicates the strength of the relationships based on standardized correlation coefficients (values provided next to the arrows; *** p < 0.001, ** p < 0.01, * p < 0.05, (*) p < 0.1)

Increasing local land-use intensity reduced bee abundance and richness in crop fields, but not their functional diversity (Table 3, Fig. 3a–c). We found no direct increase in yield with increasing local land-use intensity, regardless of the bee metric included. Land-use intensity directly reduced pollination in the models including richness and functional diversity, but not in the model including abundance. Abundance was the only bee metric that directly enhanced pollination. The percentage of arable land in the landscape did not affect bees or pollination. Pollination increased yield regardless of the bee variable included, whereas none of the bee metrics could be directly related to crop yield. All three models achieved a satisfactory fit (Abundance: RMSEA = 0.018; Richness: RMSEA = 0.044; Functional diversity: RMSEA = 0.047).

**Table 3:** Test statistics (z- and p-values) for each quantified relationship in the metaSEM for bees (Apoidea) separated by bee metrics (see Fig. 3 for standardized coefficients). Combinations of explanatory and response variables that were not included in the a priori structure are blank

Bees (n = 183)	Explanatory variable	Bee [Metric]	Pollination	Yield
Abundance	Local land-use intensity	z = − 2.063, p = 0.039	z = − 1.479, p = 0.139	z = 1.571, p = 0.116
	Arable fields %	z = 0.093, p = 0.926	z = 0.014, p = 0.989	
	Bee [Metric]		z = 2.179, p = 0.029	z = − 0.637, p = 0.524
	Ecosystem services			z = 2.884, p = 0.004
Richness	Local land-use intensity	z = − 2.190, p = 0.029	z = − 2.270, p = 0.023	z = 1.496, p = 0.135
	Arable fields %	z = − 0.818, p = 0.414	z = 0.227, p = 0.820	
	Bee [Metric]		z = 0.376, p = 0.707	z = 0.040, p = 0.968
	Ecosystem services			z = 3.247, p = 0.001
Functional diversity	Local land-use intensity	z = − 0.600, p = 0.549	z = − 2.439, p = 0.015	z = 1.565, p = 0.118
	Arable fields %	z = − 1.548, p = 0.122	z = 0.298, p = 0.766	
	Bee [Metric]		z = 0.801, p = 0.423	z = 0.026, p = 0.979
	Ecosystem services			z = 3.00, p = 0.003

### Ground beetles

**Fig. 4:**
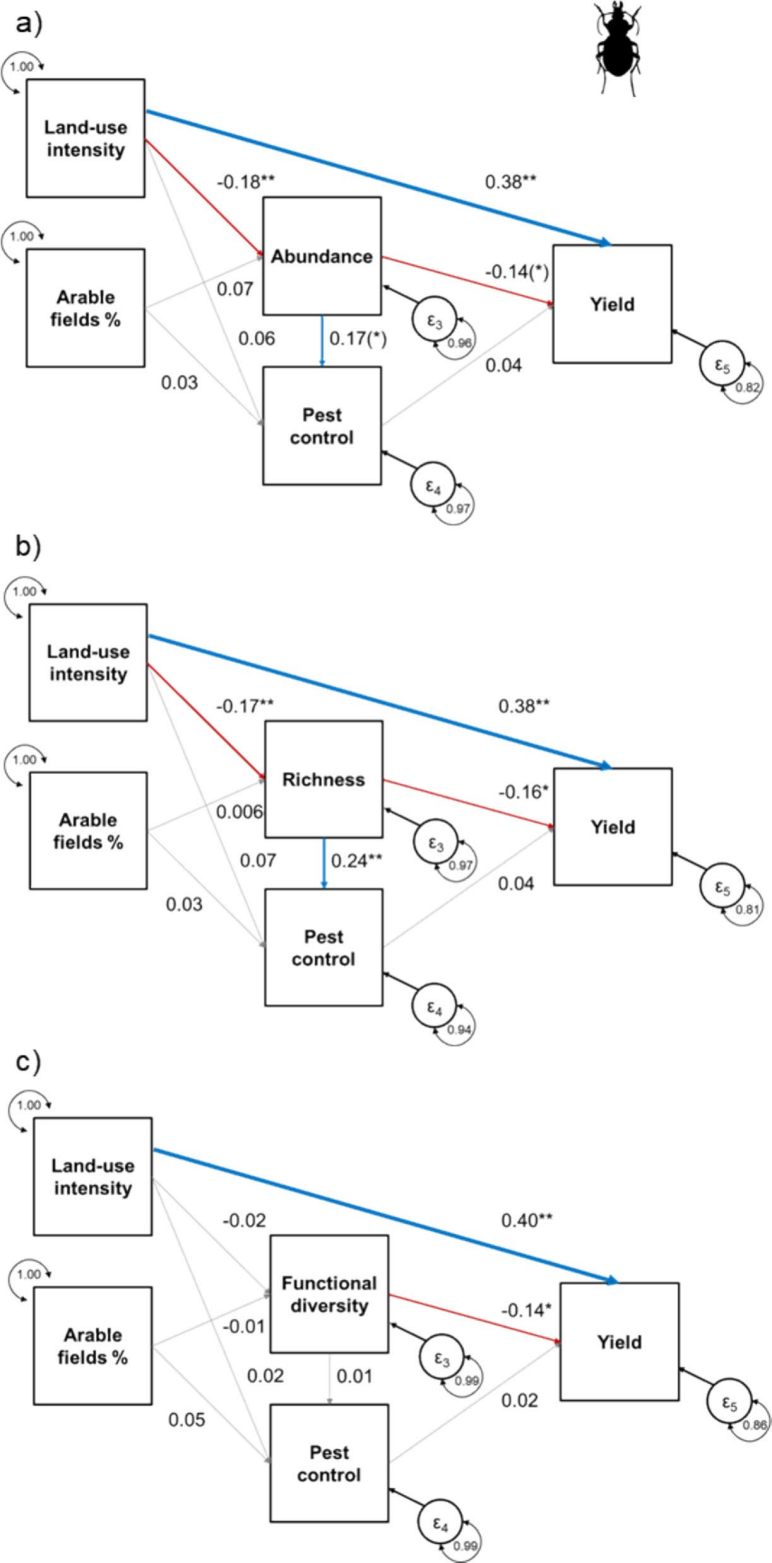
Effects of local land-use intensity and the percentage of arable land in the landscape on ground beetles. Biodiversity metrics of ground beetles include **a** Abundance, **b** Species richness, and **c** Functional diversity. The models illustrate to which extent the three calculated biodiversity metrics influence pest control and how this ecosystem service relates to crop yields. Furthermore, the models illustrate direct impacts of local land-use and the three biodiversity metrics on crop yield. Blue arrows indicate positive, red arrows indicate negative, and grey arrows indicate non-significant relationships (p > 0.1). The width of arrows indicates the strength of the relationships based on standardized correlation coefficients (values provided next to the arrows; *** p < 0.001, ** p < 0.01, * p < 0.05, (*) p < 0.1)

Increases in local land-use intensity strongly increased crop yields (Table 4, Fig. 4a–c). Similar to the model for bees, intensive land use reduced ground beetle abundance and richness, but not functional diversity. We found no direct relationship between land-use intensity and natural pest control. The percentage of arable land in the landscape was not associated with ground beetles in the field or with natural pest control. Higher ground beetle species richness was associated with increased pest control, and a higher ground beetle abundance tended to improve natural pest control. In contrast, the functional diversity of ground beetles was not related to pest control. Higher ground beetle species richness and functional diversity were associated with lower yields, and higher abundance tended to result in lower yields. We found no relationship between pest control and crop yield. All three models achieved a satisfactory fit (RMSEA < 0.001 for all models).

**Table 4:** Test statistics (z- and p-values) for each quantified relationship in the metaSEM for ground beetles (Carabidae) separated by ground beetle metrics (see Fig. 4 for standardized coefficients)

Ground beetles (n = 480)	Explanatory variable	Ground beetle [Metric]	Pest control	Yield
Abundance	Local land-use intensity	z = − 3.117, p = 0.002	z = 0.803, p = 0.422	z = 2.999, p = 0.003
	Arable fields %	z = 1.406, p = 0.160	z = 0.477, p = 0.634	
	Ground beetle [Metric]		z = 1.869, p = 0.062	z = − 1.864, p = 0.063
	Ecosystem services			z = 0.485, p = 0.628
Richness	Local land-use intensity	z = − 2.662, p = 0.008	z = 0.974, p = 0.330	z = 2.920, p = 0.004
	Arable fields %	z = 0.109, p = 0.913	z = 0.588, p = 0.556	
	Ground beetle [Metric]		z = 3.277, p = 0.001	z = − 2.360, p = 0.018
	Ecosystem services			z = 0.579, p = 0.563
Functional diversity	Local land-use intensity	z = − 0.359, p = 0.720	z = 0.335, p = 0.738	z = 3.224, p = 0.001
	Arable fields %	z = − 0.242, p = 0.809	z = 0.718, p = 0.473	
	Ground beetle [Metric]		z = 0.177, p = 0.859	z = − 2.302, p = 0.021
	Ecosystem services			z = 0.282, p = 0.778

### Spiders

**Fig. 5:**
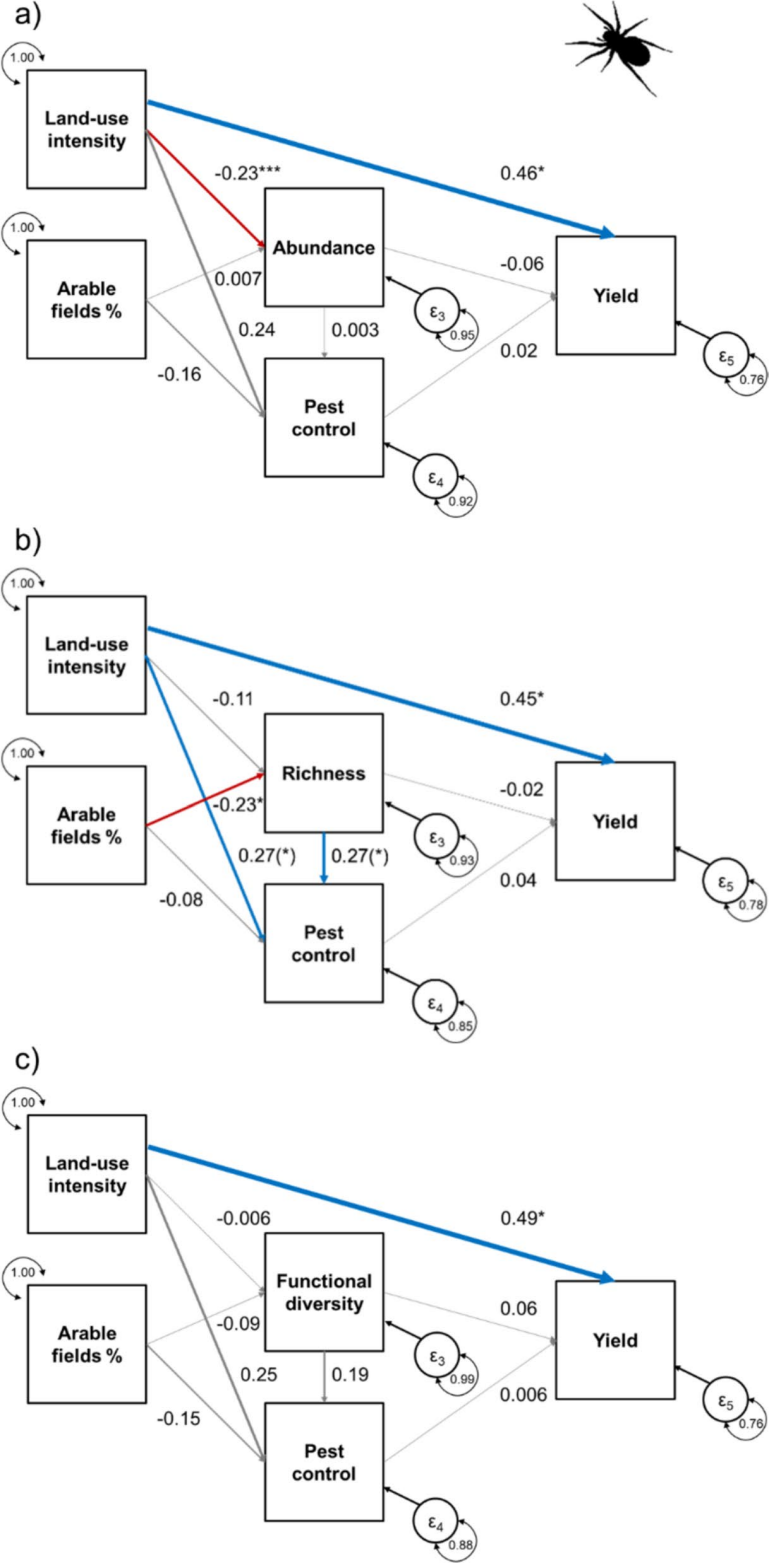
Effects of local land-use intensity and the percentage of arable land in the landscape on spiders. Biodiversity metrics of spiders include **a** Abundance, **b** Species richness, and **c** Functional diversity. The models illustrate to which extent the three calculated biodiversity metrics influence pest control and how this ecosystem service relates to crop yields. Furthermore, the models illustrate direct impacts of local land-use and the three biodiversity metrics on crop yield. Blue arrows indicate positive, red arrows indicate negative, and grey arrows indicate non-significant relationships (p > 0.1). The width of arrows indicates the strength of the relationships based on standardized correlation coefficients (values provided next to the arrows; *** p < 0.001, ** p < 0.01, * p < 0.05, (*) p < 0.1)

Local land-use intensity was the only significant predictor of crop yield, regardless of the spider metric included (Table 5, Fig. 5a–c). Local land use reduced spider abundance, but not species richness or functional diversity. Instead, spider richness, but not abundance or functional diversity, was reduced in fields with a higher proportion of arable habitats in the surrounding landscape. We found trends towards higher pest control with increasing spider species richness and local land-use intensity. We found no significant relationships between the percentage of arable habitats in the landscape and pest control, between pest control and yield, or between spider metrics and yield. All three models achieved a satisfactory fit (Abundance: RMSEA = 0.029; Richness: RMSEA = 0.052; Functional diversity: RMSEA = 0.027).

**Table 5:** Test statistics (z- and p-values) for each quantified relationship in the metaSEM for spiders (Araneae) separated by spider metrics (see Fig. 5 for standardized coefficients)

Spiders (n = 246)	Explanatory variable	Spider [Metric]	Pest control	Yield
Abundance	Local land-use intensity	z = − 3.355, p < 0.001	z = 1.486, p = 0.137	z = 2.268, p = 0.023
	Arable fields %	z = 0.084, p = 0.933	z = − 1.043, p = 0.297	
	Spider [Metric]		z = 0.020, p = 0.984	z = − 0.618, p = 0.537
	Ecosystem services			z = 0.116, p = 0.907
Richness	Local land-use intensity	z = − 1.422, p = 0.155	z = 1.676, p = 0.094	z = 2.075, p = 0.038
	Arable fields %	z = − 2.476, p = 0.013	z = − 0.485, p = 0.628	
	Spider [Metric]		z = 1.799, p = 0.072	z = − 0.179, p = 0.858
	Ecosystem services			z = 0.209, p = 0.835
Functional diversity	Local land-use intensity	z = − 0.085, p = 0.932	z = 1.607, p = 0.108	z = 2.429, p = 0.015
	Arable fields %	z = − 0.756, p = 0.449	z = − 0.952, p = 0.341	
	Spider [Metric]		z = 1.291, p = 0.197	z = 0.642, p = 0.521
	Ecosystem services			z = 0.033, p = 0.974

## Discussion

Across all three taxonomic groups, increasing local land-use intensity reduced arthropod abundance and species richness, both of which contributed to an increase in ecosystem services (i.e. pollination or pest control). Although local land-use intensity generally reduced the abundance of all three taxonomic groups when analysed separately, it is important to note that the pathway from land use to yield showed contrasting patterns between the two functional groups (i.e. pollinators vs. predators). For pollinators crop yield was primarily enhanced by the abundance of bees and the resulting increase in pollination services, while for ground beetles and spiders, crop yield was strongly influenced by land-use intensity. These results call for a more nuanced discussion of the different aspects of ecological intensification depending on the functional group and ecosystem service involved.

### Impact of local- and landscape-level land use on arthropods

Across all three taxonomic groups, local land-use intensity reduced the abundance and species richness but not functional diversity. When analysed separately, local land-use intensity reduced the abundance of all three taxonomic groups as well as the species richness of bees and ground beetles. This result is in line with many recent studies documenting declines in insect biomass during the last decades (Hallmann et al. 2017; Samu et al. 2023; Ziesche et al. 2024) and that land use is a key driver of the ongoing decline of insects (Seibold et al. 2019; Cardoso et al. 2020). In addition, land-use intensity (e.g. high fertilizer, herbicide, and insecticide inputs) reduced the richness of bees and ground beetles, likely due to direct negative effects on food resources (i.e. floral diversity, arthropods for prey; resource-mediated effects: Diehl et al. 2012; Goulson et al. 2015). Reduction in species numbers may also be the result of lower availability of specific microhabitats (structure-mediated effects: Diehl et al. 2012).

Spider species richness was the only arthropod metric that was significantly influenced by the percentage of arable land in the surrounding landscapes. Although spiders have no wings, many species are very successful long-distance dispersers via ballooning (Bonte 2012). As this is a passive process with no directional control by the spider, the local spider community can be expected to reflect the local habitat conditions but also the composition and configuration of the surrounding landscape (Schmidt et al. 2008; Bucher et al. 2010). Although foraging bees differ vastly in their mobility, many bee species pollinating crops are also using resources outside the crop fields. As such, they are affected by the structure of the surrounding landscape to provide supplementary food resources or complementary nesting sites (Jha and Kremen 2013; Redhead et al. 2016). To better understand the role of the landscape context for bees, more detailed landscape-level information related to availability of flower resources and nesting habitat would thus be needed.

Reasons for the absence of a significant relationship between local- and landscape-level land use and functional diversity in our metaSEMs across all three groups might be due to the long-term effects of agriculture resulting in homogeneous, species-poor arthropod communities in crop fields with very few disturbance-tolerant species lacking extreme trait values (i.e. shifting baseline, see also Birkhofer et al. 2015; Forrest et al. 2015). In addition, measurements of traits at the individual level would be desirable to better understand the relationship between land use and trait composition. These measures are not available in a meta-analysis that is based on the synthesis of multiple primary studies and species trait information from the literature.

### Effect of arthropods on ecosystem services and yield

Pollination was positively related to the abundance but not to the species richness or functional diversity of bees in the studied oilseed rape fields (see also Kleijn et al. 2015). In metaSEMs with species richness or functional diversity, pollination was negatively influenced by local land-use intensity. This direct effect can be explained by the indirect relationship via bee abundance not included in these models (compare Fig. 3a with Fig. 3b and Fig. 3c). The abundance of honey bees in the oilseed rape studies may overrule the role of wild bees, which contribute to increase species richness and functional diversity and can enhance pollination in the absence of honey bees (e.g. Hoehn et al. 2008). However, a global meta-analysis showed that additional wild bee species can often increase fruit set regardless of the total abundance of honey bees (Garibaldi et al. 2013). Pollination increased crop yield regardless of the bee metric included in the model. For oilseed rape, we demonstrate a full causal chain from local land-use intensity reduncing bee abundance which was in turn positively associated with pollination and yield. As the benefit of pollination for seed set was even larger than the contribution of increasing land-use intensity, suporting bee abundance via a reduction in land-use intensity can increase crop yields in oilseed rape.

The species richness of ground beetles, but not their abundance or functional diversity, enhanced pest control services. Similarly, the standardized correlation coefficient from spider species richness to pest control was even higher compared to the same relationship for ground beetles but larger variations in the relationship for spiders prevented the rejection of the null hypothesis. Our results indicate that higher species richness of ground beetles is important for reducing potential pests or weeds in crop fields regardless of their abundance or functional diversity (see also Letourneau et al. 2009). For example, even if different ground beetle species are of the same body size or prefer the same vegetation layer, they can still differ in activity period (e.g. phenology; Winqvist et al. 2011) and therefore vary in their ability to reduce pest organisms. A previous Pan-European study on the role of functional diversity of ground beetles on aphid control revealed a positive effect of functional diversity, which incorporated the same traits (Bucher et al. 2024). Incorporating more relevant traits in this meta-analysis or trait measurements at the individual level might have revealed stronger relationships between functional diversity and pest control that are potentially underlying the observed positive relationship between species richness and pest control.

Interestingly, the species richness and functional diversity of ground beetles were negatively related to crop yields. We believe that this unexpected result highlights the complexity of the relationships between predators and pest control: ground beetles can feed on beneficial arthropods and pests (Prasad and Snyder 2006; Staudacher et al. 2018). Intraguild predation is relatively common among generalist predators and can dampen top-down control of pest organisms (Finke and Denno 2003; Rusch et al. 2015). These relationships can also include alternative food sources (e.g. prey that is not a pest, but also seeds) or any other variable affecting ground beetle diversity and yield and that were not included in the available studies for our meta-analysis. Several ground beetle species (but not spiders) are granivorous or herbivorous and can benefit from crop fields in the landscape (e.g. Raderschall et al. 2022). Ground beetles in arable fields might directly reduce grain yield or harvested biomass whereas in natural habitats they would more likely engage in weed control due to the lack in grain (Rusch et al. 2016; Yvoz et al. 2021; Carbonne et al. 2023). Thus, generalist predators such as ground beetles or spiders can influence crop yields negatively or positively depending on either their food preference, the availabitity of prey types, and interactions with the habitat (see Tscharntke et al. 2016). A larger food web perspective, including specialist natural enemies, should therefore be applied to more deeply investigate these multilayer interaction networks.

### Comparison between the taxonomic groups

When comparing the cascading effects from land use to yield, differences between the taxonomic groups and associated ecosystem services are striking: higher land-use intensity reduced bee abundance and bee abundance was positively associated with pollination and crop yield in oilseed rape. Although, high land-use intensity reduced the abundance of both predatory groups and ground beetle richness, these biodiversity metrics were either positively, negatively or not significantly related to pest control or yield of wheat and barley fields. In these crops, agricultural inputs mainly determined yield, with no substantial contribution from natural pest control. Note that agricultural impact also contributed directly to pest control likely via insecticide applications (see Fig. 5b). Our comparison suggest that the contribution of service providers to crop yield differs between mutualistic and antagonistic species interactions (i.e. pollination vs. pest control).

However, such an interpretation needs to be treated with caution, as both ecosystem services were quantified at different scales: pollination, including self-/wind-pollination, was either quantified based on the seed set of individual flowers or the role of insects for pollination was quantified by comparing enclosed flowers with control flowers (i.e. presence and absence of insect pollinators). However, yield measurement at the plant or field scale can offer differing estimates (Bishop et al. 2020). Predation, on the other hand, was quantified on the basis of aphid and seed removal from cards or bite marks on dummy caterpillars as a proxy for predation by any predator (Meyer et al. 2015, Bötzl et al. 2020) and harvested grain or dry biomass at the field scale is taken as a measure of yield. A recent study on the damage of cereal leaf beetles (*Oulema* spp.) showed that damage is very heterogeneously distributed across fields: For example, grain weight decreased only by 1.6% for 75% of the plants but by 18% for 10% of the plants (Samu et al. 2024). Thus, taking the mean per field (or only having one sampling point of leaf damage per field) is more relevant for farmers but often insufficient to capture the full potential of pest control for yield. Future studies on the role of natural pest agents on crop yield should deploy a higher sampling effort in the fields to better detect the indirect effect of predators on crop plants and should consider larger taxonomic ranges for natural enemies (i.e. also considering specialist enemis such as parasitoids).

In addition to spatial scales, temporal variability in ecosystem services and crop yield are critical to uncover the contribution of biodiversity to stabilize yields (Schellhorn et al. 2015). For example, if certain species are absent in one year, others with similar trait syndromes may compensate and maintain service levels, reflecting the insurance hypothesis or portfolio effect (Loreau et al. 2001). While the most efficient trait syndromes might lead to peak performance, the resilience of ecosystem services heavily relies on complementarity among species. This dynamic is often overlooked in studies limited to a single season. Long-term studies are better suited to capturing changes in both taxonomic and functional diversity over multiple years, providing a more accurate estimation of their roles and contributions to ecosystem services.

Finally we note that our study is associative, such that in a strict sense we do not prove causality in spite of using SEMs. This calls for future studies that use experimental approaches to disentangle the separate and joint consequences of land-use intensity and effects of biodiversity on yields. Such experimental approaches would also be desirable to disentangle the role of land-use intensity and ecosystem services for crop yield in different farming systems, to reveal the extent to which an increase in land-use intensity can compensate for a loss in ecosystem services versus a reduction in land-use intensity where ecosystem services can overcompensate the resulting yield loss as suggested under the ecological intensification paradigm (Bommarco et al. 2013).

## Conclusion

Our meta-analysis based on 37 datasets quantifying the cascade from land use to crop yield via arthropods and ecosystem services resulted in contrasting patterns depending on the arthropod metric and taxonomic group but also on the way ecosystem services were quantified. Although local land-use intensity reduced the abundance of all three arthropod groups, only bee abundance enhanced pollination services and crop yield, supporting the potential of this taxonomic group for ecological intensification in oilseed rape fields. For the two generalist predatory groups, however, wheat and barley yield was primarily determined by the agricultural inputs with contrasting effects of predator metrics on pest control and yield. Understanding the role of generalist predators in pest control remains a major challenge in ecological studies. Additionaly, the potential of ecosystem service providers to increase resilience in food production and to overcompensate yield losses under ecological intensification warrants further research efforts. Our meta-analysis highlights the detrimental impact of agriculture on the abundance of arthropods across taxa. Differences in the response and effect of pollinators and predators in agroecosystems ask for a more nuanced view on ecological intensification and for experimental studies on multiple taxonomic groups and ecosystem services that apply comparable methods at similar scales to quantify the intertwined interaction between land use, ecosystem services, and food production.

## Supplementary Information

Below is the link to the electronic supplementary material.

Supplementary file1 (XLSX 155 KB)

## Data Availability

Meta-data for the included datasets and site-level land use data, biodiversity metrices, ecosystem services and yield data are provided as supplementary material.

## References

[CR1] Bengtsson J, Ahnström J, Weibull AC (2005) The effects of organic agriculture on biodiversity and abundance: a meta-analysis. J Appl Ecol 42(2):261–269

[CR2] Benisch, C (2023) kerbtier.de – Käferfauna Deutschlands. URL: https://www.kerbtier.de (08.12.2022).

[CR3] Birkhofer K, Smith HG, Weisser WW, Wolters V, Gossner MM (2015) Land-use effects on the functional distinctness of arthropod communities. Ecography 38(9):889–900

[CR4] Birkhofer, K., Buxton, M., Feng, L., Simba, L., & Diekötter, T. (2024). Conserving insects for the provision of ecosystem services. In Routledge Handbook of Insect Conservation (pp. 53–62). Routledge.

[CR5] Bishop J, Garratt MPD, Breeze TD (2020) Yield benefits of additional pollination to faba bean vary with cultivar, scale, yield parameter and experimental method. Sci Rep 10:2102 32034193 10.1038/s41598-020-58518-1 PMC7005869

[CR6] Blandenier G (2009) Ballooning of spiders (Araneae) in Switzerland: general results from an eleven-year survey. Arachnology 14(7):308–316

[CR7] Blüthgen N, Klein AM (2011) Functional complementarity and specialisation: the role of biodiversity in plant–pollinator interactions. Basic Appl Ecol 12(4):282–291

[CR8] Blüthgen N, Dormann CF, Prati D, Klaus VH, Kleinebecker T, Hölzel N, Weisser WW (2012) A quantitative index of land-use intensity in grasslands: Integrating mowing, grazing and fertilization. Basic Appl Ecol 13(3):207–220

[CR9] Boetzl FA, Konle A, Krauss J (2020) Aphid cards–Useful model for assessing predation rates or bias prone nonsense? J Appl Entomol 144(1–2):74–80

[CR10] Bommarco R, Kleijn D, Potts SG (2013) Ecological intensification: harnessing ecosystem services for food security. Trends Ecol Evol 28(4):230–238 23153724 10.1016/j.tree.2012.10.012

[CR11] Bonte, D. (2012). Cost–benefit balance of dispersal and the evolution of conditional dispersal strategies in spiders. In Spider ecophysiology (pp. 67–78). Berlin, Heidelberg: Springer Berlin Heidelberg.

[CR12] Bucher R, Herrmann JD, Schüepp C, Herzog F, Entling MH (2010) Arthropod colonisation of trees in fragmented landscapes depends on species traits. Open Ecol J. 10.2174/1874213001003010111

[CR13] Bucher R, Batáry P, Baudry J, Beaumelle L, Čerevková A, de la Riva EG, Birkhofer K (2024) Functional diversity of ground beetles improved aphid control but did not increase crop yields on European farms. Ecol Appl 34:e3035 39373261 10.1002/eap.3035 PMC11610661

[CR14] Büttner G (2014) CORINE land cover and land cover change products. Land use and land cover mapping in Europe: practices & trends. Springer, Netherlands, Dordrecht, pp 55–74

[CR15] Carbonne B, Muneret L, Laurent E, Felten E, Ducourtieux C, Henon N, Petit S (2023) Conservation agriculture affects multitrophic interactions driving the efficacy of weed biological control. J Appl Ecol 60(9):1904–1916

[CR16] Cardinale BJ, Duffy JE, Gonzalez A, Hooper DU, Perrings C, Venail P, Naeem S (2012) Biodiversity loss and its impact on humanity. Nature 486(7401):59–67 22678280 10.1038/nature11148

[CR17] Cardoso P, Pekár S, Jocqué R, Coddington JA (2011) Global patterns of guild composition and functional diversity of spiders. PLoS ONE 6(6):e21710 21738772 10.1371/journal.pone.0021710 PMC3126856

[CR18] Cardoso P, Barton PS, Birkhofer K, Chichorro F, Deacon C, Fartmann T, Samways MJ (2020) Scientists’ warning to humanity on insect extinctions. Biol Conserv 242:108426

[CR19] Cardoso, P., Pekar, S., Birkhofer, K., Chuang, A., Fukushima, C. S., Hebets, E. A., ... & Mammola, S. (2024). Ecosystem services provided by spiders. Authorea Preprints. 10.22541/au.172538631.11011603/v1

[CR20] Cheung MWL, Chan W (2005) Meta-analytic structural equation modeling: a two-stage approach. Psychol Methods 10(1):40 15810868 10.1037/1082-989X.10.1.40

[CR21] de Bello F, Lavergne S, Meynard CN, Lepš J, Thuiller W (2010) The partitioning of diversity: showing Theseus a way out of the labyrinth. J Veg Sci 21(5):992–1000

[CR22] de Bello F, Botta-Dukát Z, Lepš J, Fibich P (2021) Towards a more balanced combination of multiple traits when computing functional differences between species. Methods Ecol Evol 12:443–448

[CR23] de la Riva EG, Ulrich W, Batáry P, Baudry J, Beaumelle L, Bucher R, Čerevková A, Felipe-Lucia MR, Gallé R, Kesse-Guyot E, Rembiałkowska E, Rusch A, Seufert V, Stanley D, Birkhofer K (2023) From functional diversity to human well-being: A conceptual framework for agroecosystem sustainability. Agric Syst 208:103659

[CR24] Diehl E, Wolters V, Birkhofer K (2012) Arable weeds in organically managed wheat fields foster carabid beetles by resource-and structure-mediated effects. Arthropod-Plant Interact 6:75–82

[CR25] Emmerson, M., Morales, M. B., Oñate, J. J., Batary, P., Berendse, F., Liira, J., ... & Bengtsson, J. (2016). How agricultural intensification affects biodiversity and ecosystem services. In Advances in ecological research (Vol. 55, pp. 43–97). Academic Press.

[CR26] Farooq MO, Razaq M, Shah FM (2022) Plant diversity promotes species richness and community stability of arthropods in organic farming. Arthropod-Plant Interact 16(6):593–606

[CR27] Finke DL, Denno RF (2003) Intra-guild predation relaxes natural enemy impacts on herbivore populations. Ecol Entomol 28(1):67–73

[CR28] Forrest JR, Thorp RW, Kremen C, Williams NM (2015) Contrasting patterns in species and functional-trait diversity of bees in an agricultural landscape. J Appl Ecol 52(3):706–715

[CR29] Gagic V, Bartomeus I, Jonsson T, Taylor A, Vinqvist C, Fischer C, Slade EM, Steffan-Dewenter I, Emmerson M, Potts SG, Tscharntke T, Weisser W, Bommarco R (2015) Functional identity and diversity of animals predict ecosystem functioning better than species-based indices. Proc R Soc B 282:20142620 25567651 10.1098/rspb.2014.2620 PMC4309003

[CR30] Gagic V, Kleijn D, Báldi A, Boros G, Jørgensen HB, Elek Z, Bommarco R (2017) Combined effects of agrochemicals and ecosystem services on crop yield across Europe. Ecol Lett 20(11):1427–1436 28901046 10.1111/ele.12850

[CR31] Gámez-Virués S, Perović DJ, Gossner MM, Börschig C, Blüthgen N, De Jong H, Westphal C (2015) Landscape simplification filters species traits and drives biotic homogenization. Nat Commun 6(1):8568 26485325 10.1038/ncomms9568 PMC4634213

[CR32] Garibaldi LA, Steffan-Dewenter I, Winfree R, Aizen MA, Bommarco R, Cunningham SA, Klein AM (2013) Wild pollinators enhance fruit set of crops regardless of honey bee abundance. Science 339(6127):1608–1611 23449997 10.1126/science.1230200

[CR33] Geiger F, Bengtsson J, Berendse F, Weisser WW, Emmerson M, Morales MB, Inchausti P (2010) Persistent negative effects of pesticides on biodiversity and biological control potential on European farmland. Basic Appl Ecol 11(2):97–105

[CR34] Goulson D, Nicholls E, Botías C, Rotheray EL (2015) Bee declines driven by combined stress from parasites, pesticides, and lack of flowers. Science 347(6229):1255957 25721506 10.1126/science.1255957

[CR35] Grime JP, Hodgson JG, Hunt R (1988) Comparative plant ecology: a functional approach to common British species. Springer, Dordrecht

[CR36] Hallmann CA, Sorg M, Jongejans E, Siepel H, Hofland N, Schwan H, De Kroon H (2017) More than 75 percent decline over 27 years in total flying insect biomass in protected areas. PLoS ONE 12(10):e0185809 29045418 10.1371/journal.pone.0185809 PMC5646769

[CR37] Harrison PA, Berry PM, Simpson G, Haslett JR, Blicharska M, Bucur M, Turkelboom F (2014) Linkages between biodiversity attributes and ecosystem services: A systematic review. Ecosys Serv 9:191–203

[CR38] Hoehn P, Tscharntke T, Tylianakis JM, Steffan-Dewenter I (2008) Functional group diversity of bee pollinators increases crop yield. Proc Roy Soc b Biol Sci 275(1648):2283–2291 10.1098/rspb.2008.0405 PMC2603237 18595841

[CR39] Hooper DU, Chapin FSIII, Ewel JJ, Hector A, Inchausti P, Lavorel P, Wardle DA (2005) Effects of biodiversity on ecosystem functioning: a consensus of current knowledge. Ecol Monogr 75:3–35

[CR40] Hooper DU, Adair EC, Cardinale BJ, Byrnes JE, Hungate BA, Matulich KL, O’Connor MI (2012) A global synthesis reveals biodiversity loss as a major driver of ecosystem change. Nature 486(7401):105–108 22678289 10.1038/nature11118

[CR41] Jak S (2015) Meta-analytic structural equation modelling. Springer, Dordrecht, pp 1–88

[CR42] Jha S, Kremen C (2013) Resource diversity and landscape-level homogeneity drive native bee foraging. Proc Natl Acad Sci 110(2):555–558 23267118 10.1073/pnas.1208682110 PMC3545746

[CR43] Karp DS, Chaplin-Kramer R, Meehan TD, Martin EA, DeClerck F, Grab H, Wickens JB (2018) Crop pests and predators exhibit inconsistent responses to surrounding landscape composition. Proc Natl Acad Sci 115(33):E7863–E7870 30072434 10.1073/pnas.1800042115 PMC6099893

[CR44] Kleijn D, Snoeijing GIJ (1997) Field boundary vegetation and the effects of agrochemical drift: botanical change caused by low levels of herbicide and fertilizer. J Appl Ecol. 10.2307/24052588

[CR45] Kleijn D, Winfree R, Bartomeus I, Carvalheiro LG, Henry M, Isaacs R, Potts SG (2015) Delivery of crop pollination services is an insufficient argument for wild pollinator conservation. Nat Commun 6(1):7414 26079893 10.1038/ncomms8414 PMC4490361

[CR46] Kleijn D, Bommarco R, Fijen TP, Garibaldi LA, Potts SG, Van Der Putten WH (2019) Ecological intensification: bridging the gap between science and practice. Trends Ecol Evol 34(2):154–166 30509848 10.1016/j.tree.2018.11.002

[CR47] Letourneau DK, Jedlicka JA, Bothwell SG, Moreno CR (2009) Effects of natural enemy biodiversity on the suppression of arthropod herbivores in terrestrial ecosystems. Annu Rev Ecol Evol Syst 40(1):573–592

[CR48] Lindroth, C.H. (1985/86) The Carabidae (Coleoptera) of Fennoscandia and Denmark. Fauna Entomologica Scandinavica, 15, 1–225.

[CR49] Lompe, A. (2023) Käfer Europas. https://coleonet.de/coleo/html/impressum.htm (accessed: 08.12.2022).

[CR50] Loreau M, Naeem S, Inchausti P, Bengtsson J, Grime JP, Hector A, Wardle DA (2001) Biodiversity and ecosystem functioning: current knowledge and future challenges. Science 294(5543):804–808 11679658 10.1126/science.1064088

[CR51] Luka, H., Marggi, W., Huber, C., Gonseth, Y. & Nagel, P. (2009). Carabidae, Ecology - Atlas. - Fauna Helvetica 24. 677 pp.

[CR52] Martin EA, Dainese M, Clough Y, Báldi A, Bommarco R, Gagic V, Steffan-Dewenter I (2019) The interplay of landscape composition and configuration: new pathways to manage functional biodiversity and agroecosystem services across Europe. Ecol Lett 22(7):1083–1094 30957401 10.1111/ele.13265

[CR53] Martin AE, Collins SJ, Crowe S, Girard J, Naujokaitis-Lewis I, Smith AC, Fahrig L (2020) Effects of farmland heterogeneity on biodiversity are similar to—or even larger than—the effects of farming practices. Agric Ecosys Environ 288:106698

[CR54] Maurer, R., & Hänggi, A. (1990). Katalog der schweizerischen Spinnen. Documenta Faunistica Helvetiae 12. Centre suisse de cartographie de la faune, Basel.

[CR55] Meyer ST, Koch C, Weisser WW (2015) Towards a standardized rapid ecosystem function assessment (REFA). Trends Ecol Evol 30(7):390–397 25997592 10.1016/j.tree.2015.04.006

[CR56] Muneret L, Mitchell M, Seufert V, Aviron S, Djoudi EA, Pétillon J, Rusch A (2018) Evidence that organic farming promotes pest control. Nature Sustain 1(7):361–368

[CR57] Nentwig W, Blick T, Bosmans R, Hänggi A, Kropf C, & Stäubli A (2022) Spinnen Europas. Version 12.2022. Online https://www.araneae.nmbe.ch (accessed: 08.12.2022).

[CR58] Prasad, R. P., & Snyder, W. E. (2006). Polyphagy complicates conservation biological control that targets generalist predators. Journal of Applied Ecology, 43(2).

[CR59] QGIS (2023). QGIS Geographic Information System. QGIS Association. Version 3.30.0. URL: http://www.qgis.org

[CR60] Raderschall CA, Lundin O, Aguilera G, Lindström SA, Bommarco R (2022) Legacy of landscape crop diversity enhances carabid beetle species richness and promotes granivores. Agr Ecosyst Environ 340:108191

[CR61] Redhead JW, Dreier S, Bourke AF, Heard MS, Jordan WC, Sumner S, Carvell C (2016) Effects of habitat composition and landscape structure on worker foraging distances of five bumble bee species. Ecol Appl 26(3):726–739 27411246 10.1890/15-0546

[CR62] Rosenheim JA, Kaya HK, Ehler LE, Marois JJ, Jaffee BA (1995) Intraguild predation among biological-control agents: theory and evidence. Biol Control 5(3):303–335

[CR63] Rusch A, Birkhofer K, Bommarco R, Smith HG, Ekbom B (2015) Predator body sizes and habitat preferences predict predation rates in an agroecosystem. Basic Appl Ecol 16(3):250–259

[CR64] Rusch A, Binet D, Delbac L, Thiéry D (2016) Local and landscape effects of agricultural intensification on Carabid community structure and weed seed predation in a perennial cropping system. Landscape Ecol 31:2163–2174

[CR65] Samu F, Szita É, Botos E, Simon J, Gallé-Szpisjak N, Gallé R (2023) Agricultural spider decline: long-term trends under constant management conditions. Sci Rep 13(1):2305 36759542 10.1038/s41598-023-29003-2 PMC9911793

[CR66] Samu F, Szita É, Simon J, Cséplő M, Botos E, Pertics B, Tholt G (2024) Cereal leaf beetle (*Oulema spp*.) damage reduces yield and is more severe when natural enemy action is prevented. Crop Prot 185:106893

[CR67] Schellhorn NA, Gagic V, Bommarco R (2015) Time will tell: resource continuity bolsters ecosystem services. Trends Ecol Evol 30(9):524–530 26138384 10.1016/j.tree.2015.06.007

[CR68] Schmidt MH, Thies C, Nentwig W, Tscharntke T (2008) Contrasting responses of arable spiders to the landscape matrix at different spatial scales. J Biogeogr 35(1):157–166

[CR69] Seibold S, Gossner MM, Simons NK, Blüthgen N, Müller J, Ambarlı D, Weisser WW (2019) Arthropod decline in grasslands and forests is associated with landscape-level drivers. Nature 574(7780):671–674 31666721 10.1038/s41586-019-1684-3

[CR70] Shipley B (2016) Cause and correlation in biology: a user’s guide to path analysis, structural equations and causal inference with R. Cambridge University Press

[CR71] Staudacher K, Rennstam Rubbmark O, Birkhofer K, Malsher G, Sint D, Jonsson M, Traugott M (2018) Habitat heterogeneity induces rapid changes in the feeding behaviour of generalist arthropod predators. Funct Ecol 32:809–819 29657351 10.1111/1365-2435.13028 PMC5887929

[CR72] Tilman D, Wedin D, Knops J (1996) Productivity and sustainability influenced by biodiversity in grassland ecosystems. Nature 379:718–720

[CR73] Tscharntke T, Klein AM, Kruess A, Steffan-Dewenter I, Thies C (2005) Landscape perspectives on agricultural intensification and biodiversity – ecosystem service management. Ecol Lett 8:857–874

[CR74] Tscharntke T, Tylianakis JM, Rand TA, Didham RK, Fahrig L, Batáry P, Westphal C (2012) Landscape moderation of biodiversity patterns and processes-eight hypotheses. Biol Rev 87(3):661–685 22272640 10.1111/j.1469-185X.2011.00216.x

[CR75] Tscharntke T, Karp DS, Chaplin-Kramer R, Batáry P, DeClerck F, Gratton C, Zhang W (2016) When natural habita8t fails to enhance biological pest control—Five hypotheses. Biol Conserv 204:449–458

[CR76] Tscharntke T, Grass I, Wanger TC, Westphal C, Batáry P (2021) Beyond organic farming–harnessing biodiversity-friendly landscapes. Trends Ecol Evol 36(10):919–930 34362590 10.1016/j.tree.2021.06.010

[CR77] Turin, H. (2000) De Nederlandse Loopkevers - Verspreiding en oecologie. Nationaal Natuurhistorisch Museum Naturalis, Leiden.

[CR78] Ulrich W, Batáry P, Baudry J, Beaumelle L, Bucher R, Čerevková A, de la Riva EG, Felipe-Lucia MR, Gallé R, Kesse-Guyot E, Rembiałkowska E, Rusch A, Stanley D, Birkhofer K (2023) From biodiversity to health: Quantifying the impact of diverse ecosystems on human well-being. People and Nature 5:69–83

[CR79] Violle C, Navas ML, Vile D, Kazakou E, Fortunel C, Hummel I, Garnier E (2007) Let the concept of trait be functional. Oikos 116(5):882–892

[CR80] Winqvist C, Bengtsson J, Aavik T, Berendse F, Clement LW, Eggers S, Bommarco R (2011) Mixed effects of organic farming and landscape complexity on farmland biodiversity and biological control potential across Europe. J Appl Ecol 48(3):570–579

[CR81] Wong MKL, Guénard B, Lewis OT (2019) Trait-based ecology of terrestrial arthropods. Biol Rev 94:999–1022 30548743 10.1111/brv.12488 PMC6849530

[CR82] Yvoz S, Cordeau S, Ploteau A, Petit S (2021) A framework to estimate the contribution of weeds to the delivery of ecosystem (dis) services in agricultural landscapes. Ecol Ind 132:108321

[CR83] Ziesche TM, Ordon F, Schliephake E, Will T (2024) Long-term data in agricultural landscapes indicate that insect decline promotes pests well adapted to environmental changes. J Pest Sci 97(3):1281–1297

